# Empyema Caused by *Pseudomonas luteola*: A Case Report

**DOI:** 10.5812/jjm.10923

**Published:** 2014-07-01

**Authors:** Farid Yousefi, Saeed Shoja, Negin Honarvar

**Affiliations:** 1 Department of Infectious and Tropical Diseases, Razi Hospital, Faculty of Medicine, Jundishapur University of Medical Sciences, Ahvaz, IR Iran; 2 Health Research Institute, Infectious and Tropical Disease Research Center, Jundishapur University of Medical Sciences, Ahvaz, IR Iran

**Keywords:** Empyema, Pleural Effusion, Tuberculous Pleurisy, *Pseudomonas*

## Abstract

**Introduction:**

*Pseudomonas luteola* is an uncommon opportunistic pathogen. It is recognized as an uncommon cause of infections in underlying medical disorders. Infections caused by this microorganism are health care associated.

**Case Presentation:**

The current study isolated *P. luteola* from empyema in a patient with tuberculous pleurisy, whose susceptibility to trimethoprim-sulfamethoxazole differed from previous reports.

**Conclusions:**

*P. luteola* is resistant to TMP-SMX, but in the present case *P. luteola* was susceptible to TMP-SMX

## 1. Introduction

*Pseudomonas luteola* is an aerobic, non-spore forming, Gram negative and rod-shaped bacterium (0.8-2.5 μm). This bacterium is motile owing to the presence of one or more polar flagella. It is lactose non-fermenter and grows well on MacConkey medium (1). The optimal growth temperature is 30°C. This species of *Pseudomonas* is oxidase negative and produces distinguished yellow-pigmented colonies on MacConkey agar. It does not reduce nitrate and oxidase xylose (1, 2). It was first described by Tatum et al. (3), and was previously known as Centers for Disease Control and Prevention (CDC) group Ve-1 and *Chryseomonas luteola *(1). Analysis of 16srDNA sequences of this microorganism has suggested that the genera named *Chryseomonas*, *Flavimonas* and *Pseudomonas* were synonymous; consequently, *P. luteola *was used (4). The normal habitat of *P. luteola *is unclear, although it belongs to a group of bacteria normally found in moist environments (4). They can contaminate solutions such as distilled water, disinfectants and intravenous solutions (1). There are rare reports of infections caused by *P. luteola*.

## 2. Case Report

A 38-years-old man with clinical sign of pulmonary tuberculosis and respiratory distress was admitted to Razi of Ahvaz, Iran in September 2012. He had symptoms such as mental retardation, non-productive cough, fever, night sweating, anorexia, pleuritic chest pain and weight loss, which lasted for a month. The patients’ respiratory distress worsened during the last five days. On his physical examination, he was cachectic and there was a weak sound from the left lung. His blood test indicated the following results: No leuckocytosis, erythrocyte sedimentation rate (ESR) 120 mm/h, lactate dehydrogenase 691mg/dL and total protein 4.8 g/dL. 

All aerobic blood cultures were negative after 72 hours. He had left lung pleural effusion on chest X-Ray (left hemithorax more than 50%), and diagnostic pleural tap indicated as follows: white blood cell count 595/dL (PMN: 10%, Lymph: 90%), glucose ˂ 10 mg/dL, protein 6.1 gr/dL, LDH 4804 mg/dL and Adenosine De-aminase (ADA) 107 IU, negative smear for WBC, bacteria and culture, and positive Broncho Alveolar Lavage (BAL) for acid fast bacilli. The patient with the diagnosis of pulmonary tuberculosis and tuberculous pleurisy was put under treatment with isoniazid, rifampin, ethambutol, pyrazinamide, pyridoxine; and chest tube was inserted. Twelve days later, purulent discharge was observed in his chest tube; therefore, re-tapping was done. This time, his test results were as follows:

Appearance was turbid and WBC was 76000/dL (PMN: 99%, Lymph: 1%) many WBC and many Gram negative bacilli were observed on smear and *P. luteola *isolated and diagnosed after 48 hours. *P. luteola *was sensitive to trimethoprim-sulfamethoxazole (TMP-SMX), intermediate to gentamycin and resistant to cefepime, ciprofloxacin, norfloxacin, ampicillin, and nalidixicacid. Accordingly, the patient with the diagnosis of empyema was treated with TPM-SMX (5 mg/kg from TMP), as a result of which, his chest tube discharge decreased to 50 mL/24 hours. Then he was referred for pleural decortication.

## 3. Conclusions

Based on background, physical examination, and pleural tap, the patient had tuberculous pleurisy and empyema. Isolation of *P. luteola *from empyema demonstrated the etiological role of these bacteria. No case of empyema caused by *P. luteola *had been reported before; nevertheless previous studies showed that *P. luteola *may cause bloodstream infections associated with intravenous indwelling catheters, prosthetic valve endocarditis, pancreatitis, foreign bodies and cutaneous abscesses. Rarely, non-bacteremic cases have been reported as post neurosurgical infections, peritonitis complicating appendicitis or peritoneal dialysis catheters, fatal meningitis, femur abscess, endophthalmitis, facial cellulitis, subphrenic abscess, leg ulcer in a patient with sickle disease, and hand infection (2-16).

The use of steroids and other immunodepressive therapy, the presence of a foreign body, and postsurgical instability have been suggested to predispose to infection with *P. luteola*; therefore nosocomial infections are more frequent than community acquired ones (18). Clinical isolates of *P. luteola* are often resistant to first- and second-generation cephalosporins and tetracyclines, ampicillin, and TMP-SMX, but are susceptible to third-generation cephalosporins, mezlocillin, imipenem, aminoglycosides, and quinolones (1, 5). Contrary to the previous case reports in which *P. luteola* was resistant to TMP-SMX, in the present case *P. luteola *was susceptible to TMP-SMX and the patient showed clinical response to the treatment with TMP-SMX. Finally, when decortication was performed for the patient, treatment continued with TMP-SMX and anti-tuberculosis drugs, and the patient was discharged with relative improvement after two weeks.
